# Genetic and Pharmacological YAP Activation Induces Proliferation and Improves Survival in Human Induced Pluripotent Stem Cell-Derived Cardiomyocytes

**DOI:** 10.3390/cells12172121

**Published:** 2023-08-22

**Authors:** Thuy Anh Bui, Nicholas Stafford, Delvac Oceandy

**Affiliations:** Division of Cardiovascular Sciences, Faculty of Biology, Medicine and Health, The University of Manchester, Manchester Academic Health Science Centre, Manchester M13 9PT, UK; buithuyanh92@gmail.com (T.A.B.); nicholas.stafford@manchester.ac.uk (N.S.)

**Keywords:** induced pluripotent stem cells, iPSC-derived cardiomyocytes, Yes-associated protein, Hippo signalling pathway, cell proliferation, apoptosis

## Abstract

Cardiomyocyte loss following myocardial infarction cannot be addressed with current clinical therapies. Cell therapy with induced pluripotent stem cell-derived cardiomyocytes (iPSC-CMs) is a potential approach to replace cardiomyocyte loss. However, engraftment rates in pre-clinical studies have been low, highlighting a need to refine current iPSC-CM technology. In this study, we demonstrated that inducing Yes-associated protein (YAP) by genetic and pharmacological approaches resulted in increased iPSC-CM proliferation and reduced apoptosis in response to oxidative stress. Interestingly, iPSC-CM maturation was differently affected by each strategy, with genetic activation of YAP resulting in a more immature cardiomyocyte-like phenotype not witnessed upon pharmacological YAP activation. Overall, we conclude that YAP activation in iPSC-CMs enhances cell survival and proliferative capacity. Therefore, strategies targeting YAP, or its upstream regulator the Hippo signalling pathway, could potentially be used to improve the efficacy of iPSC-CM technology for use as a future regenerative therapy in myocardial infarction.

## 1. Introduction

The human adult myocardium has an extremely limited capability for self-renewal. Consequently, myocyte loss following ischaemia can often lead to the development of heart failure. Stem cell-based replacement therapies have been considered as a potential strategy to overcome this, as stem cells have the ability to differentiate, proliferate, and thus supply the diseased heart with a source of new cardiomyocytes [1]. Human induced pluripotent stem cell-derived cardiomyocytes (iPSC-CMs) are a particularly attractive cell type for this purpose, as they provide a potentially autologous source of iPSC-CMs for transplantation, which may reduce the risk of immunological rejection [2].

Successful reprogramming of human somatic cells into iPSCs was first achieved in 2007 using a combination of four transcription factors, Oct3/4, Sox2, Klf4, and c-Myc [3]. Soon afterwards, methods for directing iPSC differentiation towards a cardiac lineage were developed [4], providing a human cardiac model with a wide range of potential in vitro and in vivo applications. hiPSC-CMs have since become a model system for drug toxicity testing, while the ability to derive patient-specific cardiomyocytes offers significant advantages when studying inherited cardiac disorders [5]. Furthermore, by providing a virtually unlimited supply of human cardiomyocytes, iPSC-CM technology could lead to significant advancements in the field of regenerative medicine.

A range of pre-clinical studies, from rodents to non-human primates, have shown iPSC-CM administration to improve cardiac function following infarction [6,7]. However, engrafted cell survival rates are typically low given the anoxic and fibrotic environment into which they must be incorporated, whilst instances of transient ventricular arrhythmia have also been noted as likely due to the immature phenotype presented by the induced cardiomyocytes [8]. The need to refine and improve upon current iPSC-CM technology is, therefore, apparent before they are ready to translate into mainstream clinical use.

A number of strategies have been explored thus far, aimed at improving iPSC-CM maturity and engraftment rates. These include incorporation into patches or cell sheets; co-administration alongside complimentary cell types, such as endothelial cells, vascular smooth muscle cells and mesenchymal stem cells; electrical and mechanical preconditioning; and genetic and/or pharmacological manipulation [9,10,11,12,13,14,15].

As a major regulator of cardiomyocyte proliferation, migration, and survival, targeting the Hippo pathway and its transcriptional co-activator YAP represents one such strategy. The Hippo pathway is a kinase cascade which, when its core mammalian Ste20-like (MST) and large tumour suppressor (LATS) kinases are active, retains YAP in its inactive cytosolic form [16]. However, during cardiac development the Hippo signal is suppressed, leading to YAP nuclear translocation and a proliferative, anti-apoptotic state [17,18]. It has been shown that both Hippo silencing and YAP activation can re-introduce adult cardiomyocytes into this proliferative state and instigate regeneration of the damaged myocardium [19,20,21,22]. As such, using a similar approach may also enhance the survival and proliferative capacity of iPSC-CMs.

In this study, we investigated the effects of two different strategies to target the Hippo/YAP cascade in iPSC-CMs. In the first approach, we inducibly overexpressed constitutively active YAP using a Tet-On system, whilst in the second approach we pharmacologically inhibited Hippo signalling using the MST1/2 inhibitor XMU-MP1 [23]. In both instances, iPSC-CM proliferation was stimulated, and survival in response to oxidative stress was improved. Interestingly, however, the two approaches exhibited subtle differences in their effects on myocyte morphology and maturation, indicating that the timing and nature of Hippo targeting could be important factors when considering any future therapeutic strategies.

## 2. Materials and Methods

### 2.1. Human Induced Pluripotent Stem Cell Culture and Differentiation

Blood cord-derived human induced pluripotent stem cells (hiPSCs) were obtained from Life Technologies (Episomal human iPSC line, A18944, Warrington, UK). hiPSC colonies were expanded on Geltrex (Gibco, Warrington, UK) coated plates in Essential 8 cell culture medium (Thermo Scientific, Warrington, UK) until at least passage 20 before differentiation.

Chemically-defined differentiation to cardiomyocytes was achieved following a previously described protocol [24]. Briefly, when ~90% confluent hiPSCs were stimulated with differentiation medium containing RPMI 1640 + HEPES + GlutaMax + B27 supplement—insulin (Invitrogen, Warrington, UK), along with 6 µM GSK3 inhibitor Chir99021 (Sigma, Gillingham, UK), for 48 h. The media were then replaced with a differentiation medium containing 2 µM Wnt inhibitor C59 (Selleckchem, Houston, TX, USA) for a further 48 h. Cells were maintained in differentiation medium from days 4 to 8, before changing to a maintenance medium (RPMI/HEPES/GlutaMax/B27 + insulin supplement) once spontaneously-beating colonies were established for prolonged culture. Cardiomyocytes were selected through the addition of 5 mM sodium lactate (Sigma, Gillingham, UK) to the media from day 15, before plating for individual assays.

### 2.2. Generation of Inducible Constitutively Active YAP Expression System

Inducible expression of constitutively active YAP was achieved using a Tet-On dual adenoviral transduction system. AdTRE S127A YAP adenoviral vector was generated by subcloning mutant human YAP^S127A^ cDNA from the pCMV-flag S127A YAP, which was a gift from Kunliang Guan (Addgene plasmid #27370; http://n2t.net/addgene:27370; RRID: Addgene_27370) [25], into pTREtight2 vector backbone containing tight TRE promoter (pTREtight2 was a gift from Markus Ralser, Addgene plasmid #19407; http://n2t.net/addgene:19407; RRID:Addgene_19407). The resulting TRE-S127A YAP fragment was cloned to pENTR11 shuttle vector (Invitrogen) to enable the generation of adenovirus construct (AdTRE-S127A YAP) using the Gateway system (Invitrogen, Warrington, UK). The reverse tetracycline-regulated transactivator (rtTA) cDNA fragment was obtained from pMA2640 plasmid, which was a gift from Mikhail Alexeyev (Addgene plasmid #25434; http://n2t.net/addgene:25434; RRID:Addgene_25434) [26]. The rtTA cDNA fragment was cloned to the pENTR11 vector before sub-cloning to the adenovirus vector as above.

### 2.3. Activation of YAP

To induce the expression of constitutively active YAP^S127A^, hiPSC-CMs were transduced with AdTREYAP and AdrtTA adenoviruses for 24 h. Cells were washed, and then treated with doxycycline (Sigma, Gillingham, UK) at concentrations ranging from 50 to 1000 ng/mL to induce YAP^S127A^ expression for a further 24 h prior to evaluation in subsequent assays. To pharmacologically activate YAP, hiPSC-CMs were treated with 1–3 µM MST1/2 inhibitor XMU-MP-1 (Selleckchem, Houston, TX, USA) [23] dissolved in DMSO for 24 h prior to assay.

### 2.4. YAP Luciferase Assay

YAP activity was assessed by luciferase reporter assay as previously described [27,28]. Gal4-TEAD4 was a gift from Kunliang Guan (Addgene plasmid #24640; http://n2t.net/addgene:24640; RRID:Addgene_24640) and pUAS-luc2 was a gift from Liqun Luo (Addgene plasmid #24343; http://n2t.net/addgene:24343; RRID:Addgene_24343) [29]. The constructs were cloned to adenovirus vector pAd-CMV-DEST (Invitrogen, Life Technologies, Warrington, UK) using a Gateway cloning system. iPSC-CMs were transduced with AdGal4-TEAD/UAS for 24 h, and then induced with YAP^S127A^ or treated with XMU-MP-1 for a further 24 h before assessing YAP activity using a Luciferase Assay System (Promega, Southampton, UK).

### 2.5. Immunocytochemistry

In preparation for immunofluorescent microscopy, cells were first washed in PBS and then fixed with 4% PFA for 15 min, before permeabilisation with 0.1% Triton X-100 for a further 10 min. Cells were then blocked in 0.5% BSA in PBS for 1 h at room temperature before proceeding to assay-dependent antibody staining. Antibody labelling was achieved through incubation with primary antibodies at a dilution of 1:100 in 0.5% BSA blocking buffer overnight at 4 °C, before incubation with fluorescent labelled secondary antibodies (1:100 in blocking buffer) for 1.5 h at room temperature the following day, unless otherwise stated. Nuclear staining was conducted through incubation with DAPI (Thermo Scientific) for 5 min prior to mounting with Vectashield mounting media (Vector Laboratories, Kirtlington, UK). Images were acquired using a Zeiss Axio Imager Snapshot Fluorescent microscope in the University of Manchester Bioimaging facility.

For assessment of iPSC differentiation to cardiomyocytes, cells were incubated with primary antibodies against Connexin 43 (Abcam, ab63851, Cambridge, UK) and cardiac troponin T (Santa Cruz Biotechnology, sc-20025; 1:50, Heidelberg, Germany), followed by Alexa-488-conjugated anti-mouse or rabbit secondary antibody (Jackson ImmunoResearch, 715-545-150/111-545-144, Ely, UK). For morphometric analysis, cells were fixed 24 h post YAP induction, stained with Alexa-488 phalloidin (Thermo Scientific, A12379), and analysed using Image J software version 1.53.

For proliferation analysis, cells were fixed 24 h following YAP induction and incubated with Alexa Fluor 488-conjugated rabbit anti pHH3 antibody (Cell Signalling Technology, #3465, Leiden, The Netherlands) or rabbit anti Ki-67 antibody (Abcam, ab15580) followed by Alexa-488-conjugated anti-rabbit secondary. EdU incorporation was assessed using an Alexa-488 ClickIT EdU Imaging Kit (Life Technologies) as per the manufacturer’s guidelines and following protocols as previously described [27].

### 2.6. YAP Nuclear Translocation Assay

To assess subcellular localisation of YAP, hiPSC-CMs were transduced with adenovirus-expressing GFP-YAP construct for 24 h prior to YAP induction, using methods as previously described [30]. Cells were fixed 24 h post-YAP induction before counterstaining with DAPI and imaging. 

### 2.7. Apoptosis Induction and Analysis

At 24 h post-YAP induction, cells were treated with 150 µM H_2_O_2_ (Sigma) for 4 h to induce apoptosis, before proceeding to fixation. Apoptosis was assessed via TUNEL assay using an In Situ Cell Death Detection Kit (Roche, Welwyn Garden City, UK) following the manufacturer’s protocol.

### 2.8. Western Blotting

Protein was extracted from cell cultures using 1% Triton Lysis Buffer and run on 10–15% SDS-PAGE gels before transfer to nitrocellulose membranes using standard protocols. Membranes were blocked in 3–5% BSA or 5% non-fat milk, before incubation with primary antibodies to YAP (Santa Cruz Biotechnology, sc376830; 1:500, Heidelberg, Germany), Oct4 (CST2840), active YAP (CST29495), Mob1a (CST13730), pMob1a (CST8699) and HRP-linked β-actin (CST12262) (all Cell Signalling Technology, 1:1000). Following incubation with horseradish peroxidase-labelled secondary antibodies (Cell Signalling, 7074/7076; 1:5000) signal was detected using enhanced chemiluminescence (GE Healthcare, Hatfield, UK) in a ChemiDoc™ XRS+imaging system (Biorad, Kidlington, UK).

### 2.9. Quantitative Real-Time Polymerase Chain Reaction

RNA was extracted from cell cultures 24 h or 7 days post-YAP induction for assessment of YAP target gene activation or cardiomyocyte maturation, respectively. Total RNA was extracted using Trizol reagent (Invitrogen) as per the manufacturer’s instructions, and DNase was treated using DNase 1 (Thermo Scientific) and quantified with a Nanodrop 8000 spectrophotometer (Thermo Scientific). A total of 2 µg of RNA was converted to cDNA using a high-capacity cDNA reverse transcription kit (Thermo Scientific), following which, qPCR was conducted using Brilliant III Ultrafast SYBR green (Agilent, Cheadle, UK) on a 7500 Fast RT-PCR system (Applied Biosystems, Warrington, UK). mRNA was quantified relative to the housekeeping gene GAPDH using the 2^−ΔΔCT^ method [31]. Primer sequences used are listed in Table 1.

### 2.10. Data Analysis

Data are presented as mean ± the standard error from the mean and were analysed using GraphPad Prism Software version 9.0.1. Statistical analysis was conducted using one- or two-way ANOVAs where appropriate, with Tukey’s post-hoc multiple comparison analysis. *p* values <0.05 were considered statistically significant.

## 3. Results

### 3.1. YAP Activity Is Diminished during iPSC Differentiation to Cardiomyocytes

**Figure 1:**
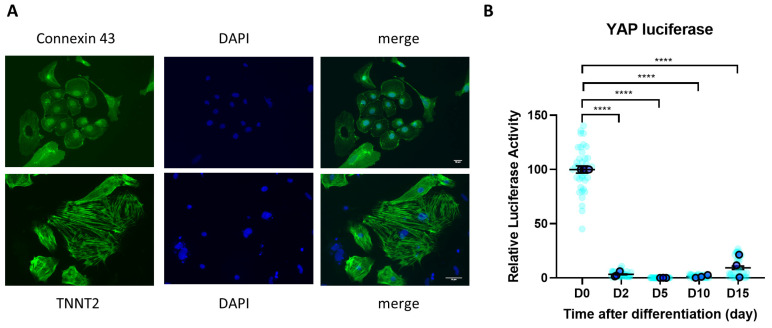
YAP activity during iPSC-CM differentiation. (**A**) iPSC-CMs expressed cardiomyocyte markers cardiac Troponin T (TNNT2 (green)) and Connexin 43 (green) following differentiation, as detected by immunocytochemistry. Scale bars represent 50 µm. (**B**) YAP activity determined by luciferase reporter assay during hiPSC differentiation to cardiomyocytes. Statistical significance determined by one-way ANOVA followed by Tukey’s post hoc multiple comparison. n = 36 replications (light blue) from N = 3 independent batches of differentiation (dark blue). **** *p* < 0.0001.

We differentiated human induced pluripotent stem cells (iPSC) into iPSC-derived cardiomyocytes (iPSC-CMs) in monolayer culture using GSK3 and Wnt inhibitors [32]. Markers of cardiomyocytes, such as cardiac Troponin T and Connexin 43, were detected in iPSC-CMs at the end of the differentiation protocol (Figure 1A). Our differentiation protocol consistently resulted in a high proportion of cardiomyocytes, with a substantial percentage exceeding 90% of cTNT-positive cells, following the successful differentiation process from iPSCs. We did not characterise whether the yield has atrial, ventricular or nodal properties; however, given that the iPSC-CMs in the yield were beating, it is likely that there was a mixture of ventricular/atrial cells with the nodal cells in the yield. 

To examine the involvement of the Hippo/YAP pathway during the process of iPSC differentiation to iPSC-CMs, we monitored the level of YAP activity using a YAP-luciferase reporter system [28]. We delivered the luciferase reporter system to iPSCs using adenovirus and measured the signal from day 0 up to day 15 of the differentiation process. We found that YAP activity, which is known as the main downstream effector of the Hippo pathway, was almost completely abolished from day 2 post differentiation (Figure 1B), indicating a strong involvement of this pathway in this process.

### 3.2. Cell Proliferation Is Reduced during iPSC Differentiation to iPSC-CM

**Figure 2:**
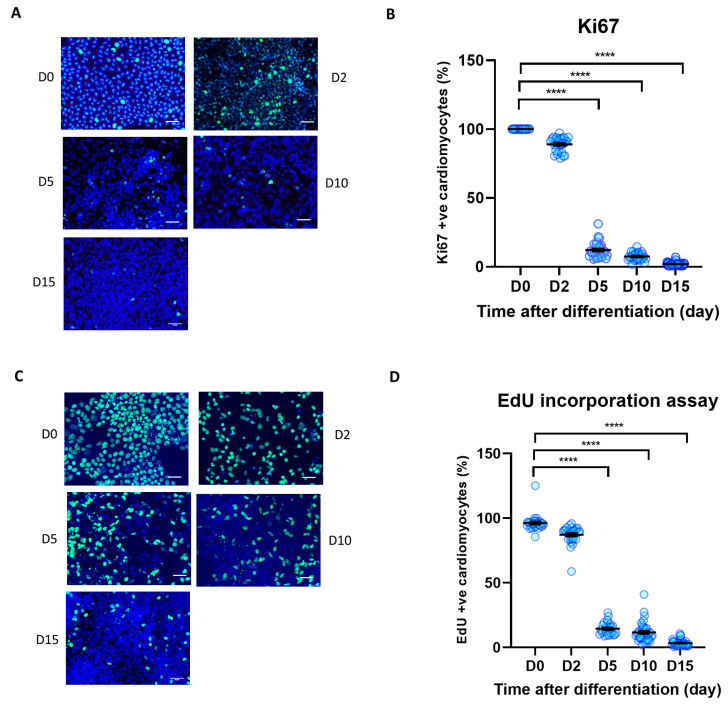
Cell proliferation during iPSC-CM differentiation. Immunocytochemistry analysis of proliferation markers during human iPSC differentiation to cardiomyocytes. (**A**,**B**) Ki67 positive nuclei (green) as a percentage of total nuclei (blue), (**C**,**D**) EdU positive nuclei (green) as a percentage of total nuclei (blue). Scale bars represent 50 µm. Statistical significance determined by one-way ANOVA followed by Tukey’s post hoc multiple comparison. n = 26–38 replications per group. **** *p* < 0.0001.

The Hippo/YAP pathway tightly modulates cell proliferation and survival, with YAP activity being directly correlated with cell proliferation rate [33]. To analyse iPSC proliferation rate during differentiation to iPSC-CMs, we conducted time-course analysis of cell proliferation markers Ki67 expression and EdU incorporation rate. As shown in Figure 2A–D, we observed a strong reduction of both Ki67 positive nuclei and EdU incorporation in iPSCs at day 5 after the start of the differentiation process. The iPSC-CM proliferation rate remained low up to day 15 post-differentiation. This indicates that undifferentiated iPSCs displayed a very high proliferation rate (~>96%), that reduced to ~3% once the cells differentiated to cardiomyocytes.

### 3.3. Re-Activation of YAP Activity Using an Inducible Expression System

**Figure 3:**
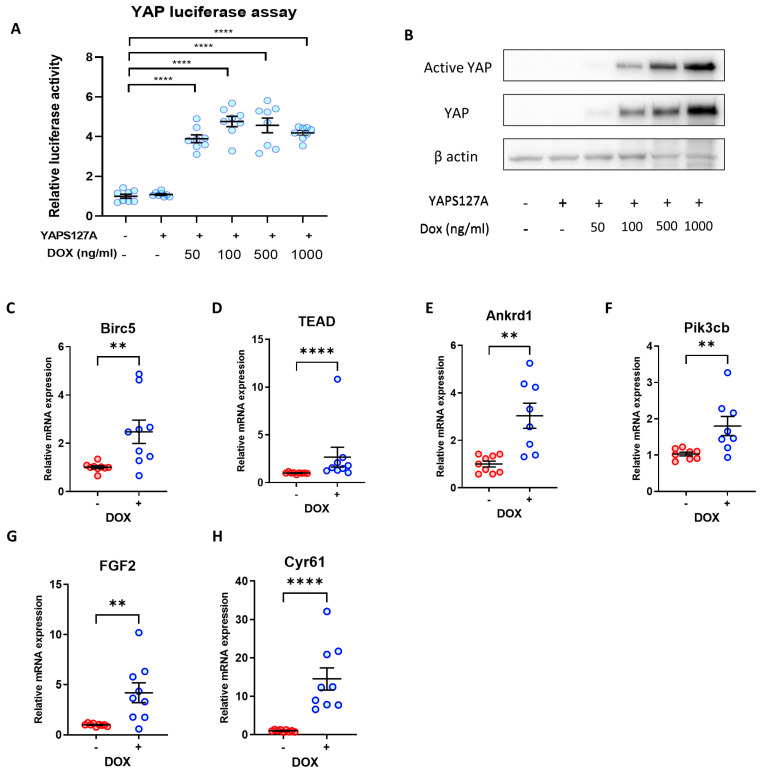
Doxycycline-induced YAPS127A expression increases YAP activity in iPSC-CMs. (**A**) YAP activity determined by luciferase reporter assay in hiPSC-CMs following transduction with TRE-YAP^S127A^ + rtTA adenovirus, and YAP induction with 50–1000 ng/mL doxycycline. Statistical significance determined by one-way ANOVA followed by Tukey’s post hoc multiple comparison. n = 8 replicates per group, **** *p* < 0.0001. (**B**) Western Blot demonstrating overexpression of YAP and active YAP in hiPSC-CMs following YAP^S127A^ transduction and induction with 50–1000 ng/mL doxycycline. (**C**–**H**) qRT-PCR analysis of transcript levels of YAP targets *Birc5*, *Tead1*, *Ankrd1*, *Pikcb3*, *Fgf2,* and *Cyr61* relative to housekeeping gene *Gapdh* 7 days following doxycycline-induced YAP^S127A^ overexpression. Statistical significance determined by unpaired *t*-test. N = 6 independent experiments, with n = 9–12 replicates in each group, ** *p* < 0.01, **** *p* < 0.0001.

We next sought to understand if re-activation of YAP in fully differentiated iPSC-CMs will restore their proliferative capacity. Using a Tet-On system, we successfully induced expression of constitutively active YAP (YAP^S127A^) in iPSC-CMs. We used an adenoviral system to achieve efficient delivery of the TRE-YAP^S127A^ and CMV-rtTA constructs to iPSC-CMs. We then treated iPSC-CMs carrying both constructs with increasing concentrations of doxycycline. As expected, we observed strong expression of the YAP^S127A^ as well as increased YAP-luciferase activity following doxycycline treatment at doxycycline doses above 500 ng/mL (Figure 3A,B). Based on these findings, we used a dose of 500 ng/mL doxycycline for further experiments.

We detected expression of known YAP target genes to examine YAP transcriptional activity in response to YAP^S127A^ overexpression. As shown in Figure 3C–H expression of *Birc5*, *TEAD1*, *Ankrd1*, *Pik3cb*, *Fgf2,* and *Cyr61* were significantly elevated following doxycycline treatment. 

### 3.4. Induction of YAP Increases iPSC-CM Proliferation

**Figure 4:**
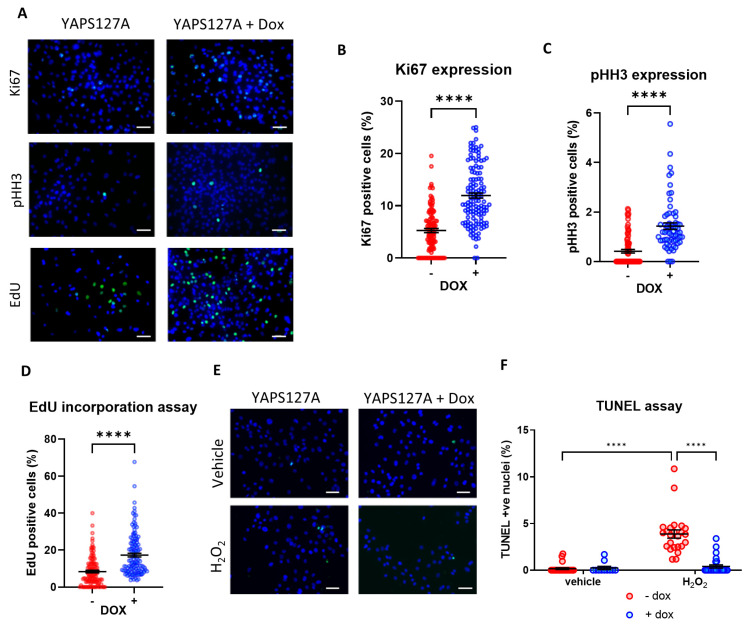
Doxycycline-induced YAP^S127A^ expression enhances iPSC-CM proliferation and survival. Immunocytochemistry analysis of proliferation markers 24 h following doxycycline-induced YAP^S127A^ overexpression in hiPSC-CMs. (**A**) Representative images of analysis of proliferation markers (Ki67 and pHH3) and EdU incorporation assay 24 h following doxycycline-induced YAP^S127A^ overexpression in iPSC-CMs. Quantification of (**B**) Ki67 positive nuclei (green), (**C**) pHH3 positive nuclei (green) and (**D**) EdU positive nuclei (green), as a percentage of total nuclei (blue). Scale bars represent 50 µm. Statistical significance determined by unpaired *t*-test. N = 3 independent batches of differentiation, with n = 111–130 replicates (Ki67), n = 64–76 replicates (pHH3) and n = 119–133 replicates (EdU) per group. **** *p* < 0.0001. (**E**,**F**) Apoptosis induced by 4 h exposure to 150 µM H_2_O_2_ in hiPSC-CMs, following 24 h of doxycycline-induced YAP^S127A^ overexpression. Results expressed as TUNEL positive nuclei (green) as a percentage of total nuclei (blue). Scale bars represent 50 µM. Statistical significance determined by two-way ANOVA and Tukey test for post hoc multiple comparison. n = 11–27 replicates per group. **** *p* < 0.0001.

To assess if inducible expression of YAP^S127A^ affects iPSC-CM proliferation we analysed expression of markers of cell cycle entry: Ki67 and phospho-Histone H3 (pHH3). As expected, we observed a significant increase in both Ki67 and pHH3 positive nuclei in cells bearing the inducible construct following doxycycline treatment (Figure 4A–C), suggesting more YAP^S127A^ overexpressing cells entering the cell cycle compared to controls. In addition, we also tested the DNA synthesis rate in these cells by performing an EdU incorporation assay. Consistent with the analysis of markers of cell cycle entry, YAP^S127A^ expression increased EdU incorporation (Figure 4A,D), suggesting an increase in DNA synthesis. Together, these data suggest a potential pro-proliferative effect of inducible YAP activation in iPSC-CMs.

### 3.5. YAP Activation Protects iPSC-CMs against Apoptosis 

Since the Hippo pathway and YAP have been known to play a significant role in the regulation of cell death and apoptosis, we examined the level of apoptosis in iPSC-CMs expressing active YAP in response to oxidative stress due to H_2_O_2_ treatment. As shown in Figure 4E,F, we observed a significant reduction in H_2_O_2_-mediated apoptosis levels once YAP^S127A^ expression was induced by doxycycline.

### 3.6. Expression of Cardiomyocyte Maturation Markers following YAP Activation in iPSC-CMs 

As YAP is a transcriptional co-activator that is associated with embryonic development [17], we next questioned whether activation of YAP would affect iPSC-CM maturation. We therefore performed qPCR analysis to detect the expression of genes that have been associated with cardiomyocyte maturation, such as the ryanodine receptor (RyR2), sarcoplasmic reticulum calcium pump (SERCA2), myosin light chain 2 (Myl2), connexin 43 (Cx43), alpha myosin heavy chain (Myh6), and beta myosin heavy chain (Myh7).

**Figure 5:**
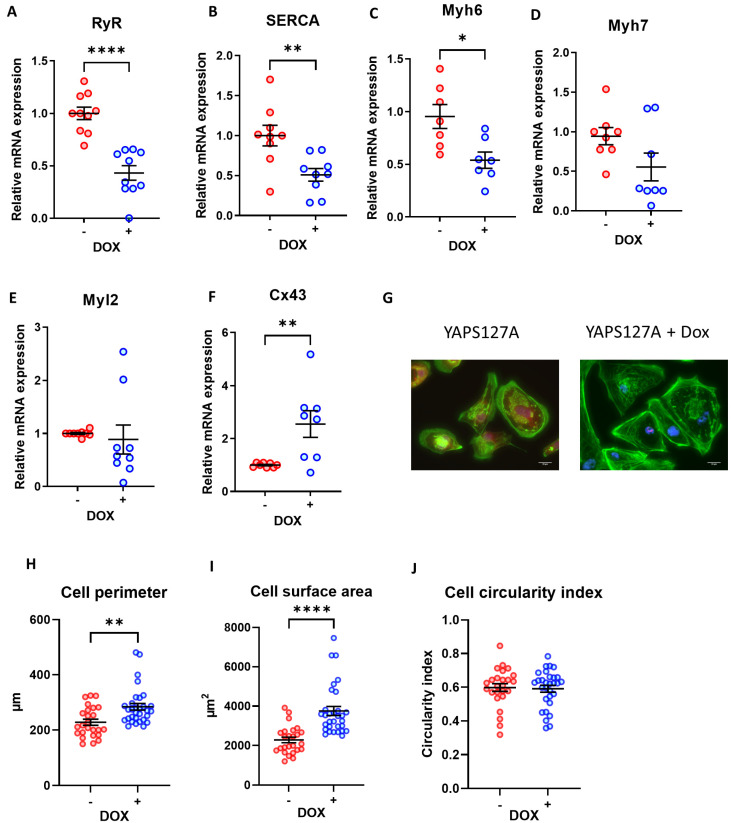
iPSC-CM maturation and morphology following doxycycline-induced YAP^S127A^ expression. (**A**–**F**) qRT-PCR analysis of transcript levels of cardiomyocyte maturation markers *RyR*, *SERCA2A*, *Myh6*, *Myh7*, *Myl2,* and *Cx43* relative to housekeeping gene *Gapdh* 7 days following doxycycline-induced YAP^S127A^ overexpression. Statistical significance determined by unpaired *t*-test. N = 6 independent experiments, with n = 9–12 replicates in each group, * *p* < 0.05, ** *p* < 0.01, **** *p* < 0.0001. (**G**–**J**) iPSC-CMs fixed and stained with phalloidin (green) 7 days following doxycycline-induced YAP^S127A^ overexpression for analysis of cell perimeter, cross-sectional area and circularity. Scale bars represent 20 µM. Statistical significance determined by unpaired *t*-test. n = 25–30 replicates per group. ** *p* < 0.01, **** *p* < 0.0001.

We found a marked reduction in most genes tested (with the exception of Cx43) in iPSC-CMs with induced expression of YAP^S127A^ (Figure 5A–F). This indicates a reduction in maturity following YAP activation in iPSC-CMs.

### 3.7. Morphological Analysis of iPSC-CMs after Induction of YAP Activity

We went on to assess the morphological features of iPSC-CMs following YAP^S127A^ overexpression. As presented in Figure 5G, the phalloidin staining of cardiomyocyte sarcomeres following YAP^S127A^ + Dox treatment reveals a highly disorganised structure, indicating an immature state or the possible initiation of cell cycle entry in the myocytes. Additionally, induction of YAP^S127A^ expression significantly enhanced cell size and cell perimeter (Figure 5G–I) suggesting a pro-growth effect of YAP^S127A^ in iPSC-CMs. However, the circularity index, which is measured based on area/perimeter^2^, was not affected following YAP induction (Figure 5J).

### 3.8. Re-Activation of YAP Activity in iPSC-CMs Using MST1 Inhibitor (XMU-MP-1)

**Figure 6:**
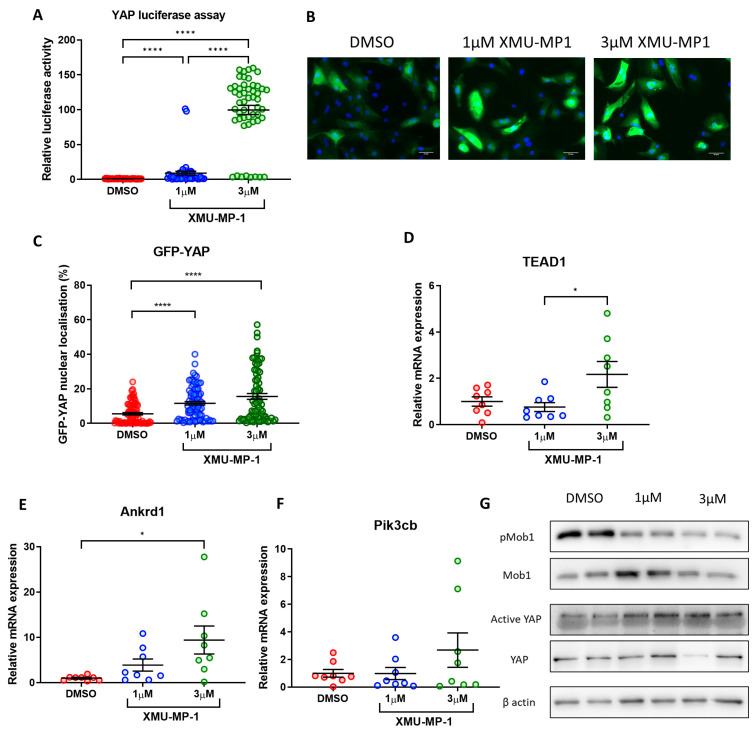
MST inhibitor XMU-MP-1 increases YAP activity in iPSC-CMs. (**A**) YAP activity determined by luciferase reporter assay in hiPSC-CMs following 24 h treatment with 1–3 µM XMU-MP-1. Statistical significance determined by one-way ANOVA and Tukey test for post hoc multiple comparison. N = 5 independent batches of differentiation, with n = 47–51 replicates per group. **** *p* < 0.0001. (**B**,**C**) Immunocytochemistry analysis of GFP-YAP nuclear translocation following 24 h XMU-MP-1 treatment. Scale bars represent 50 µM. Statistical significance determined by one-way ANOVA and Tukey test for post hoc comparison. N = 3 independent batches of differentiation, with 76–83 replicates per group. **** *p* < 0.0001. (**D**–**F**) qRT-PCR analysis of transcript levels of YAP targets *Tead1*, *Ankrd1,* and *Pikcb3* relative to housekeeping gene *Gapdh* 7 days following XMU-MP-1 treatment. Statistical significance determined by one-way ANOVA and Tukey test for post hoc comparison. N = 4 independent experiments, with n = 8 replicates in each group. * *p* < 0.05. (**G**) Western blot analysis of Mob1 phosphorylation and active YAP levels in iPSC-CMs 24 h post XMU-MP-1 treatment.

Our data above provide strong evidence that re-activation of YAP using a genetic approach in fully differentiated iPSC-derived cardiomyocytes might increase cell proliferation and survival. This may facilitate the establishment of more expandable and durable iPSC-CM colonies, which may improve their suitability for use as a cell-based therapy. To understand if this can also be achieved using a pharmacological approach, we used XMU-MP-1, a potent inhibitor of the key Hippo pathway components MST1/2. Accumulating evidence has shown that treatment with XMU-MP-1 increases YAP activity in various cell types including primary cardiomyocytes [23,30]. Following treatment with 1–3 µM XMU-MP-1, we observed a dose dependent increase in YAP activity in fully differentiated iPSC-CMs as indicated by YAP-luciferase assay (Figure 6A). Consistently, YAP nuclear translocation (Figure 6B,C) as well as expression of several YAP target genes (*Tead1*, *Ankrd1*, *Pikcb3*—Figure 6D–F) were also increased following treatment with XMU-MP-1. Western blot analysis revealed that phosphorylation of MOB1, a main downstream target of MST1/2, was downregulated (Figure 6G). Consistently, there was an upward trend in active YAP levels upon exposure to increasing doses of XMU-MP-1. Together, our data demonstrate that XMU-MP-1 can successfully induce YAP activity in iPSC-CMs.

### 3.9. Treatment with XMU-MP-1 Increases iPSC-CM Proliferation and Improves Survival

**Figure 7:**
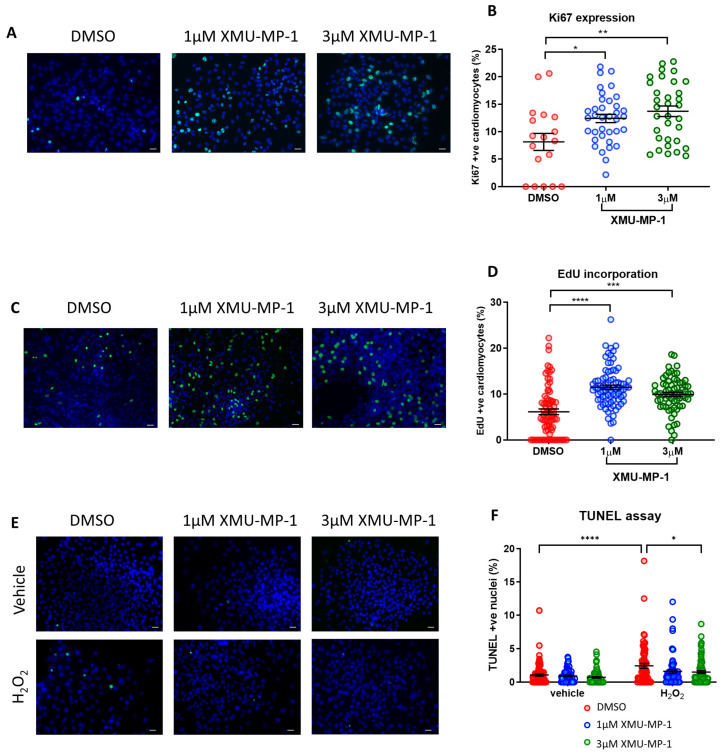
MST inhibitor XMU-MP-1 enhances iPSC-CM proliferation and survival. Immunocytochemistry analysis of proliferation markers 24 h following XMU-MP-1 treatment in hiPSC-CMs. (**A**,**B**) Ki67 positive nuclei (green) and (**C**,**D**) EdU positive nuclei (green), as a percentage of total nuclei (blue). Scale bars represent 50 µM. Statistical significance was determined by one-way ANOVA and Tukey test for post hoc comparison. N = 3 independent batches of differentiation, with n = 18–36 replicates (Ki67) and n = 67–80 replicates (EdU) per group. * *p* < 0.05, ** *p* < 0.01, *** *p* < 0.001, **** *p* < 0.0001. (**E**,**F**) Apoptosis induced by 4 h exposure to 150 µM H_2_O_2_ in hiPSC-CMs, co-treated with XMU-MP-1. Results expressed as TUNEL positive nuclei (green) as a percentage of total nuclei (blue). Scale bars represent 50 µM. Statistical significance determined by two-way ANOVA and Tukey test for post hoc comparison. N = 4 batches of differentiation, with n = 69–90 replicates per group. * *p* < 0.05, **** *p* < 0.0001.

We next performed experiments to evaluate iPSC-CM proliferation rate following treatment with XMU-MP-1. As shown in Figure 7A–D we observed a significant increase in cell proliferation, as indicated by enhanced expression of the mitotic marker Ki67 and an elevation of EdU incorporation in cells treated with XMU-MP-1. This corroborates our data using the inducible YAP^S127A^ overexpression system, confirming the idea that YAP/Hippo modulation can induce iPSC-CM proliferation. Moreover, we also found that XMU-MP-1 treatment reduced apoptosis following oxidative stress as suggested by TUNEL assay (Figure 7E,F).

### 3.10. Effects of XMU-MP-1 Treatment on iPSC-CM Maturation and Morphology

**Figure 8:**
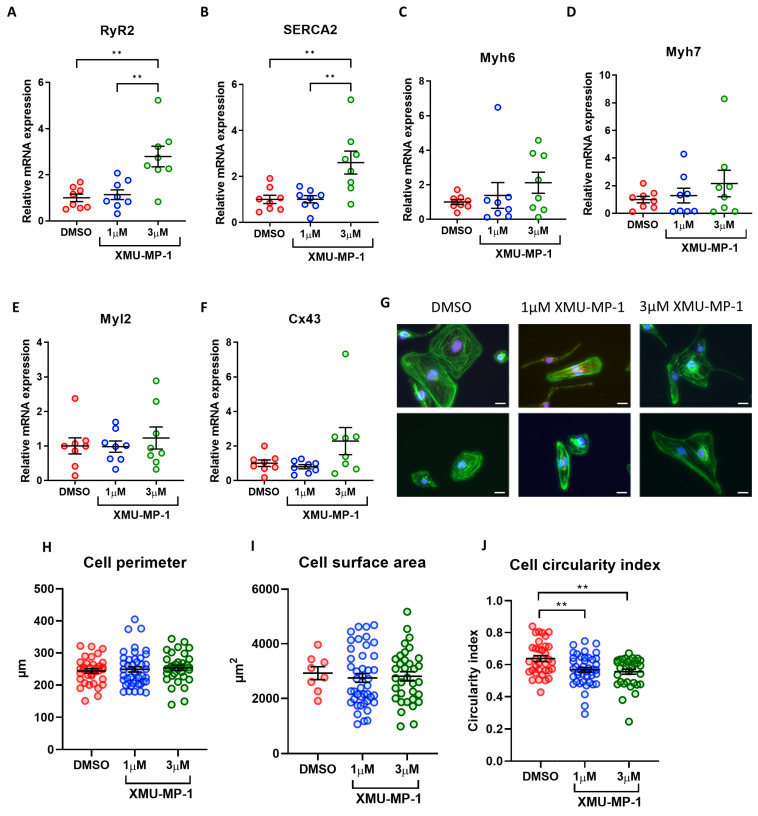
iPSC-CM maturation and morphology following XMU-MP-1 treatment. (**A**–**F**) qRT-PCR analysis of transcript levels of cardiomyocyte maturation markers *RyR*, *SERCA2A*, *Myh6*, *Myh7*, *Myl2,* and *Cx43* relative to housekeeping gene *Gapdh* 7 days following XMU-MP-1 treatment. Statistical significance determined by one-way ANOVA and Tukey test for post hoc multiple comparison. N = 4 batches of differentiation, with n = 8 replicates in each group. ** *p* < 0.01. (**G**–**J**) iPSC-CMs fixed and stained with phalloidin (green) 7 days following XMU-MP-1 treatment for analysis of cell perimeter, cross-sectional area and circularity. Scale bars represent 20 µM. Statistical significance determined by one-way ANOVA and Tukey test for post hoc multiple comparison. n = 8–43 replicates per group. ** *p* < 0.01.

To assess the effects of XMU-MP-1 treatment on iPSC-CM maturation we detected expressions of genes related to cardiomyocyte maturation. Interestingly, contrary to the data from the inducible YAP^S127A^ overexpression model, we detected increased expression of calcium-handling genes such as SERCA2 and RyR2 (Figure 8A,B). On the other hand, we found no changes in the expression of genes encoding for myofilament proteins such as myosin light chain 2, and alpha- and beta-myosin heavy chain (Figure 8C–F). This again was in contrast to the YAP inducible model, wherein we observed reduced expression of these genes.

We then analysed the morphology of the iPSC-CMs. We found that there were no significant changes in cell perimeter or cell area after XMU-MP-1 treatment (Figure 8G–I). On the other hand, we observed a slight reduction in circularity index, indicating enhanced cell elongation (Figure 8J). Overall, our data indicated that treatment with XMU-MP-1 did not reduce expression of cardiomyocyte maturation markers nor increase cardiomyocyte size.

## 4. Discussion

The present study provides evidence that YAP activation, through both a genetic approach and pharmacological targeting of the Hippo signalling pathway, improves iPSC-CM proliferation and survival. Interestingly, we also present data which suggest that iPSC-CM maturation and morphology may be affected differently depending on whether YAP is induced genetically or pharmacologically.

Cell-based therapy has long been considered a promising avenue for regenerative medicine, including as a treatment option in the diseased myocardium. This is particularly the case following acute myocardial infarction and in the failing heart. Clinical trials have indeed found that adult stem cell therapies are safe in MI and HF patients [34], and are largely associated with improvements in left ventricular ejection fraction [34,35]. However, no improvements in mortality have been found to date, and data suggest that the beneficial effects of adult stem cell therapies occur through their paracrine release of protective factors rather than through their engraftment and differentiation into new cardiomyocytes [36]. In fact, preclinical studies in mice have shown mesenchymal stem cell engraftment rates to be as low as 0.44% following injection into the left ventricle, while intracoronary transfer rates into human infarcted myocardia are similarly low at 1.3–2.6% [37,38]. Hence, the number of surviving transplanted myocytes pales in comparison to the ~1 billion cells that may be lost following ischaemia. Consequently, there is a need to improve upon current cell-based therapies, and iPSCs already differentiated into cardiomyocytes could help address previous issues.

iPSC-CMs have consistently shown beneficial effects when implanted into the infarcted myocardium, leading to improved cardiac function and attenuated remodelling in rodent and porcine models [6,39] whilst also enhancing contractility upon transplantation into non-human primates following myocardial infarction [7]. Importantly, iPSC-CMs share similar electrophysiological and neurohormonal characteristics with resident cardiomyocytes, and are capable of engrafting and forming gap junctions with them [40]. However, a number of challenges still remain if they are to prove an effective translational therapy for human heart disease, including improving reprogramming efficiency, proliferative capacity and survival upon implantation [2]. Moreover, differentiated iPSC-CMs present with an immature phenotype compared to adult cardiomyocytes, and this may be responsible for the transient induction of ventricular arrhythmias, especially in primates [7,41]. Thus, strategies to enhance the technology are paramount.

One major finding in our study is the discovery that activating YAP in iPSC-CMs can significantly enhance cell proliferation and cell survival in response to stress signals. First, our iPSC-CM differentiation method aligns with the majority of other studies focused on the large-scale expansion of cardiomyocytes generated from iPSCs, which achieved an impressive over 250-fold amplification [42]. Furthermore, our protocol has yielded a considerable population of iPSC-CMs (cTNT > 90%), as observed in another study that aimed to produce iPSC-CMs on a large scale for examination in a big animal model of myocardial infarction [43,44]. In addition, our study has demonstrated that YAP activation has the potential to enhance the survival capability of stressed iPSC-CMs in vitro. This effect can be attributed to the ability of active YAP to initiate a transcriptional programme within stressed cardiomyocytes, leading to the expression of antioxidant genes through YAP interaction with FoxO1 or the TEAD/Pitx complex [45,46]. Nonetheless, the potential of YAP activation for iPSC-CM survival in vivo remains to be investigated. Previous studies have suggested several methods to stimulate the regenerative potential of native cardiomyocytes or stem cells in the damaged heart, but satisfactory outcomes have not been achieved so far [47]. If the re-activation of YAP activity within iPSC-CMs in vivo could enhance endogenous cardiomyocytes or stem cell-based regeneration, it would present a novel approach to improve iPSC-CM survival within implanted hearts to overcome the major hurdle of poor survival and integration of transplanted iPSC-CMs within host tissues.

The observed beneficial effects of YAP activation on enhancing iPSC-CM proliferation and survival can potentially be attributed to the manipulation of the Hippo signalling pathway. The Hippo pathway regulates heart size during embryonic development, with inactivation through Sav deletion leading to increased proliferation and overgrown hearts [48], whilst YAP deletion impairs cardiogenesis and leads to embryonic lethality in mice [17]. In this study, we examined YAP activity during the differentiation process from pluripotency into cardiomyocytes and found a sharp decline in the first days of differentiation which was sustained throughout the process. This suggests that once cell fate has been directed towards cardiomyocytes, the Hippo pathway is activated and YAP activity is consequently downregulated, similar to the situation in the post-neonatal and adult heart [19]. However, it has been shown that the YAP inhibitor verteporfin prevents cardiac differentiation of hiPSCs, keeping cells in an early cardiac progenitor cell-like state, indicating that at least a baseline level of YAP activity may be required for cardiac differentiation [49]. Interestingly, our observed reduction in YAP activity also correlated with reduced proliferation in iPSC-CMs during the differentiation process. Therefore, this prompted us to hypothesise that reactivating YAP in iPSC-CMs may be able to stimulate a proliferative phenotype.

The pivotal role of YAP in governing cell cycle progression and stem cell pluripotency has been extensively documented. Firstly, YAP functions as a crucial regulator of the cell cycle, controlling the expression of essential genes involved in DNA replication, mitosis, and checkpoint progression [50,51]. Mechanistically, YAP translocates to the nucleus and interacts with transcription factors such as TEAD and AP1 to stimulate the expression of target cell cycle regulators [51,52]. Additionally, YAP can directly regulate cardiomyocyte proliferation, and consequently heart size, through its interaction with other signalling pathways such as the Wnt/β-catenin pathway [48]. Notably, the application of mechanical strain on epithelial cells induces rapid nuclear translocation of YAP, leading to β-catenin nucleus accumulation and transcriptional activation, thus facilitating prompt cell cycle entry [48,53]. Moreover, YAP plays a significant role in regulating the self-renewal and differentiation potential of stem cells. Studies have demonstrated that transient expression of exogenous YAP can drive the conversion of somatic cells into a tissue-specific stem/progenitor cell state [54]. YAP has also been reported to regulate epidermal stemness through its interaction with the Notch signalling pathway [55]. These findings align with our study, which revealed that transient activation of YAP^S127A^ induces proliferative phenotypes and immaturity in iPSC-CMs.

Indeed, we provide strong evidence that both inducible overexpression of constitutively active YAP and pharmacological activation via MST1/2 inhibitor XMU-MP1 stimulate proliferation in iPSC-CMs. Moreover, both strategies also proved effective at improving iPSC-CM survival when challenged with oxidative stress. This indicates that both genetic and pharmacological approaches towards YAP activation could provide potential therapeutic advantages for iPSC-CMs. In contrast, we also observed some phenotypic differences between YAP-overexpressing cells and XMU-MP1 treated cells. Overexpression of YAP^S127A^ appeared to reduce the expression of a number of cardiomyocyte maturation markers, including contractile proteins and sarcoplasmic reticulum calcium transporters, whilst increasing overall cell size. In contrast, XMU-MP1 treatment increased the expression of SERCA2A and RYR2, whilst not affecting myofilament gene expression, indicating limited effects on cardiomyocyte maturation, whilst not affecting the cell size. It is not clear why the two approaches have different effects in terms of myocyte maturation and morphology, but it is quite possible that MST inhibition could affect multiple pathways in addition to Hippo/YAP signalling, such as FOXO and Akt pathways [56]. 

Another crucial aspect of cardiomyocyte maturation involves changes in the expression of calcium handling proteins and the transition of cardiomyocyte metabolic status from glycolysis to fatty acid oxidation [57]. We acknowledge the limitations of our study, particularly regarding the examination of calcium handling solely at the mRNA level, without presenting the protein expression levels of these genes in both models of YAP activation. However, it is also important to note that changes in mRNA level may be detected sooner than protein due to the protein’s half-life. Therefore, we believe that in this study, where the effects of YAP induction were analysed at a relatively short time point following overexpression (24 h), analysis of maturity gene expression using qPCR method is acceptable to provide evidence of changes of gene expression.

Additionally, we did not investigate the metabolic status of hiPSC-CMs under YAP activation, as it was beyond the scope of this study. However, the Hippo pathway and YAP have been reported to play a significant role in upregulating cellular glycolysis in response to energy supply and stress signals. For example, active YAP, particularly under conditions of energy stress release, can promote glycolysis by upregulating the transcription of GLUT3, a glucose transporter [58]. Moreover, in specific cellular contexts, such as in breast cancer cells, YAP can enhance glycolytic activity to meet energy demands for cancer cell proliferation via activating the Hedgehog pathway and inducing the expression of glycolysis-promoting factors such as HK2 and PFKFB3 [59]. Activation of YAP in stressed cardiomyocytes, such as under conditions of pressure overload, has been demonstrated to enhance the binding of YAP to the GLUT1 proximal promoter alongside TEAD1 and HIF-1α [60]. This interaction stimulates the transcription of the GLUT1 gene in cardiomyocytes, subsequently inducing glycolysis and shifting the metabolic preference from fatty acid metabolism as part of cardiac hypertrophic responses. It would be intriguing to explore whether YAP^S127A^ activation or XMU-MP1 treatment could also shift iPSC-CM metabolism from fatty acid oxidation towards glycolysis. This metabolic alteration, reminiscent of foetal cardiac metabolism, could be assessed alongside the examination of changes in the expression of cardiomyocyte maturation markers, as mentioned above. 

This study adds to a growing arsenal of strategies which may enhance iPSC-CM efficacy. For example, maturation has been shown to be improved following co-culture with mesenchymal stem cells [11], while chemical manipulation of a number of pathways via GSK3, P38 MAPK or CAMK2 inhibitors can stimulate iPSC-CM proliferation [15]. Specifically, targeting Hippo pathway and YAP activation appears to hold great promise in this area. Both siRNA-mediated YAP silencing and the YAP inhibitor verteporfin have been shown to reduce iPSC-CM proliferation [61]. Meanwhile, Hippo inhibition via XMU-MP1 in combination with YAP activator sphingosine-1-phosphate, the YAP-TEAD activator TT-10 alone, or YAP overexpression, all lead to increased cell counts [62,63,64]. Moreover, YAP activity appears integral to cardiomyocytes’ ability to regain cell cycle re-entry [64]. Stimulation of proliferation and survival appear to be the main effects of YAP activation and/or Hippo inhibition, with less reported effects on maturation and functionality. YAP activation via TT-10 was found to not alter contraction or maturity of iPSC-CMs [62], whilst calcium transients have been shown to not be impacted by YAP siRNA [63]. Therefore, manipulation of Hippo signalling in iPSC-CMs appears to hold promise as a means of stimulating proliferation, without affecting the functionality.

In vitro, enhancing YAP activity in iPSC-CMs may, therefore, be of significant benefit, through increasing cellular proliferation and survival rates and thus enabling the production of larger populations of human cardiomyocytes for use in applications such as drug discovery and toxicity testing. It remains to be seen whether Hippo-modulated iPSC-CMs are capable of improving engraftment rates and proliferating when injected in vivo, however, especially in the harsh environments encountered during myocardial ischaemia or heart failure. Future studies will need to assess this, as well as the best way to manipulate Hippo signalling in the cells either genetically, pharmacologically and/or temporally. Optimisation of the delivery and timing will be key to avoid unwanted effects, such as the potential for teratoma formation.

## 5. Conclusions

In conclusion, YAP activation in iPSC-CMs enhances their proliferative capacity and survival in response to stress, whether induced via a genetic or pharmacological approach. Consequently, the Hippo/YAP pathway presents a prime target to develop strategies which could improve the efficacy of iPSC-CM technology to treat ischaemia and heart failure in the future.

## Figures and Tables

**Table 1:** qPCR primer sequences used. Forward and reverse primers were used at a final concentration of 1 µM.

Gene Symbol	Forward Primer (5′-3′)	Reverse Primer (5′-3′)
*hPik3cb*	TATTTGGACTTTGCGACAAGACT	TCGAACGTACTGGTCTGGATAG
*hTead1*	ATGGAAAGGATGAGTGACTCTGC	TCCCACATGGTGGATAGATAGC
*hAnkrd1*	AGTAGAGGAACTGGTCACTGG	TGTTTCTCGCTTTTCCACTGTT
*hCyr61*	GGTCAAAGTTACCGGGCAGT	GGAGGCATCGAATCCCAGC
*hBirc5*	AGGACCACCGCATCTCTACAT	AAGTCTGGCTCGTTCTCAGTG
*hFgf2*	AGAAGAGCGACCCTCACATCA	CGGTTAGCACACACACTCCTTTG
*hCtgf*	CAGCATGGACGTTCGTCTG	AACCACGGTTTGGTCCTTGG
*hMyh6*	GCCCTTTGACATTCGCACTG	GGTTTCAGCAATGACCTTGCC
*hMyh7*	CTTTGCTGTTATTGCAGCCATT	AGATGCCAACTTTCCTGTTGC
*hMyl2*	TTGGGCGAGTGAACGTGAAAA	CCGAACGTAATCAGCCTTCAG
*hSerca2a*	ATGGGGCTCCAACGAGTTAC	TTTCCTGCCATACACCCACAA
*hCx43*	TGGTAAGGTGAAAATGCGAGG	GCACTCAAGCTGAATCCATAGAT
*hRyr2*	GGCAGCCCAAGGGTATCTC	ACACAGCGCCACCTTCATAAT
*hGapdh*	GGATTTGGTCGTATTGGG	GGAAGATGGTGATGGGATT

## Data Availability

All data generated or analysed during this study are included in this published article.
